# Prevention and Management Strategies for Diabetic Neuropathy

**DOI:** 10.3390/life12081185

**Published:** 2022-08-03

**Authors:** Sasha Smith, Pasha Normahani, Tristan Lane, David Hohenschurz-Schmidt, Nick Oliver, Alun Huw Davies

**Affiliations:** 1 Section of Vascular Surgery, Department of Surgery and Cancer, Imperial College London, London W6 8RF, UK; sasha.smith@imperial.ac.uk (S.S.); p.normahani@imperial.ac.uk (P.N.); tristan.lane@imperial.ac.uk (T.L.); 2 Imperial Vascular Unit, Imperial College Healthcare NHS Trust, London W6 8RF, UK; 3 Department of Vascular Surgery, Cambridge University Hospitals NHS Foundation Trust, Cambridge CB2 0QQ, UK; 4 Pain Research Group, Department of Surgery and Cancer, Imperial College London, London SW10 9NH, UK; d.hohenschurz-schmidt19@imperial.ac.uk; 5 Section of Metabolic Medicine, Department of Metabolism, Digestion and Reproduction, Imperial College London, London W2 1PG, UK; nick.oliver@imperial.ac.uk; 6 Division of Medicine and Integrated Care, Imperial College Healthcare NHS Trust, London W2 1NY, UK

**Keywords:** diabetes, diabetic neuropathy, distal symmetrical polyneuropathy, glycemic control, lifestyle modifications, footcare, diagnostic evaluation, pain management, neuromodulation, nutraceuticals

## Abstract

Diabetic neuropathy (DN) is a common complication of diabetes that is becoming an increasing concern as the prevalence of diabetes rapidly rises. There are several types of DN, but the most prevalent and studied type is distal symmetrical polyneuropathy, which is the focus of this review and is simply referred to as DN. It can lead to a wide range of sensorimotor and psychosocial symptoms and is a major risk factor for diabetic foot ulceration and Charcot neuropathic osteoarthropathy, which are associated with high rates of lower limb amputation and mortality. The prevention and management of DN are thus critical, and clinical guidelines recommend several strategies for these based on the best available evidence. This article aims to provide a narrative review of DN prevention and management strategies by discussing these guidelines and the evidence that supports them. First, the epidemiology and diverse clinical manifestations of DN are summarized. Then, prevention strategies such as glycemic control, lifestyle modifications and footcare are discussed, as well as the importance of early diagnosis. Finally, neuropathic pain management strategies and promising novel therapies under investigation such as neuromodulation devices and nutraceuticals are reviewed.

## 1. Introduction

Diabetes is a major global health problem affecting half a billion people worldwide. Its global prevalence is rising at an alarming rate and has been forecast to reach 700 million by 2045 [1]. Diabetic neuropathy (DN) is an important and common complication of diabetes, with a lifetime prevalence of more than 50% among people with diabetes [2]. DN can encompass several patterns of neuropathy, owing to the numerous possible sites of nerve damage. This review will focus on the most prevalent and studied type, distal symmetrical polyneuropathy, which will be referred to as DN throughout. 

DN is an insidious and often disabling disease. Sensory symptoms are diverse, ranging from numbness to dysesthesia, pain and allodynia, and typically begin in the feet and spread proximally. Motor function can also be affected, resulting in weakness, atrophy, gait disorder and loss of coordination, preventing patients from engaging in activities of daily living. More recently, the considerable psychosocial and quality of life (QoL) impacts of DN have been recognized [3]. Furthermore, DN is a major risk factor for diabetic foot ulceration and Charcot neuropathic osteoarthropathy (Charcot foot), which are independent risk factors for lower limb amputation and mortality [4]. The associated economic burden is high; in the United Kingdom (UK), the annual cost of managing DN exceeds GBP 300 million [5], with further foot complications expected to cost an additional GBP 1 billion [6]. 

The pathophysiology of DN is characterized by peripheral nerve fiber and microvessel dysfunction. This is primarily driven by hyperglycemia and other metabolic factors, such as hyperlipidemia and impaired insulin signaling, which lead to a variety of downstream pathogenic pathways. In particular, hyperglycemia leads to overactivation of the polyol, glycation, protein kinase C, poly (ADP-ribose) polymerase (PARP) and hexosamine pathways, all of which contribute to oxidative stress in nerves and microvessels. The complexities of these pathways are beyond the scope of this article, but we refer the reader to relevant recent review articles [7,8,9].

These pathogenic pathways influence nerve structure and function. The myelin sheath is disrupted, and Schwann cells dissociate from both myelinated and unmyelinated nerve fibers, produce less neurotrophic factors and undergo apoptosis [9,10,11,12,13]. Then, axonal transport and signaling are affected [9,14,15,16], potentially at the axo-glial interface [9,17], resulting in axonal loss. This occurs first in unmyelinated and small thinly myelinated fibers, and then large myelinated fibers [9,10,18]. The mechanisms underlying axonal loss in DN are unknown, but signals may originate in the dorsal root ganglia or the spinal cord [9,19,20].

In the closely connected microvasculature, there are changes in basement membrane density, pericyte function, endothelial cell function and the formation of arteriovenous shunts occur, all signifying ischemic damage [9,21,22,23,24,25,26]. This reduces angiogenic factors, such as vascular endothelial growth factor, which are neuroprotective [9,27]. The severity of microangiopathy has been linked to DN on multiple occasions, including outcomes such as nerve conductivity [9,21,24,28,29,30]. Despite these advances, the pathophysiology of DN remains largely unknown, limiting the development of pathogenetic treatments [9].

Instead, national and international clinical guidelines recommend several prevention and management strategies for DN based on the best available evidence. Prevention strategies aim to address DN before symptoms develop, or to prevent the progression of DN, whereas management strategies treat symptoms of DN that patients already have. These guidelines focus on prevention through glycemic control, lifestyle modifications and footcare, all of which can be difficult to achieve for a variety of reasons. For people with painful DN, management through pharmacotherapies is recommended; however, this is frequently sub-optimal and does not target the approximately 70% of people with DN who do not experience pain [2,31]. The importance of early diagnosis of DN and how it may allow for advanced implementation of strategies to prevent disease progression has been emphasized, though there are safety concerns with current invasive diagnostic evaluation tools, so research must focus on developing noninvasive alternatives. Novel therapies are in development, such as neuromodulation devices and nutraceuticals; however, progress has been hindered by the limited understanding of DN pathogenesis and a decline in industry-invested research [9,32].

The aim of this article is to provide a narrative review of DN prevention and management strategies. First, the epidemiology of DN and its risk factors will be briefly summarized, discussing differences between diabetes subtypes. The diverse clinical manifestations will be described, with a focus on sensorimotor and psychosocial symptoms, and further foot complications. Prevention strategies recommended by clinical guidelines will be discussed and the diagnostic evaluation of DN will be briefly reviewed. Finally, neuropathic pain management strategies and promising novel therapies under investigation will be discussed.

## 2. Epidemiology

Previous research has suggested that the prevalence of DN is higher in people with type 2 diabetes (T2DM) than in people with type 1 diabetes (T1DM) [33]. This has been reflected in several large studies. For example, in the SEARCH for Diabetes in Youth Study, there was a significant difference in the prevalence of DN among adolescents with T2DM as compared to those with T1DM (26% vs. 8%, respectively) [34]. The ACCORD trial, a landmark randomized trial assessing the effect of glycemic treatments on microvascular complications, reported DN in 42% (4345/10,201) of people with T2DM at baseline [35]. This is substantially higher as compared to the Diabetes Control and Complications Trial (DCCT), which reported DN in only 6% of people with T1DM at baseline [36,37]. However, comparisons between studies should be made with caution, as reported prevalence rates vary greatly due to differences in methodology and study populations [38]. In the ACCORD trial, for example, the average duration of diabetes at baseline was ten years, compared to six years in the DCCT. In addition, DN was determined using the Michigan Neuropathy Screening Instrument, whereas clinical evidence and nerve conduction studies were used in the DCCT [35,36,37].

These pioneering studies also identified risk factors for DN. In the DCCT and Epidemiology of Diabetes Interventions and Complications (EDIC) studies, the T1DM cohort (n *=* 1441) were followed-up for 14 years. The cases of DN had increased significantly from 6% at baseline to 30% at final follow-up, implicating age, duration of diabetes and chronic hyperglycemia as risk factors [36,37]. A recent review of DCCT/EDIC data validated these risk factors, as well as height, macroalbuminuria, pulse rate, beta-blocker use and sustained albuminuria [32]. The Pittsburgh Epidemiology of Diabetes Complications study, which enrolled 400 participants with T1DM supports some of these findings; 18% of 18- to 29-year-olds were found to have DN, compared to 58% of people ≥ 30 years old [39]. Similar trends have also been observed in other longitudinal studies conducted in Europe and Africa [40,41]. Additional cardiometabolic risk factors include dyslipidemia, hypertension and central obesity, which may explain the reports of higher DN prevalence in people with T2DM [42,43]. Future studies may consider stratification of other diabetes subtypes, termed by the World Health Organization as “hybrid” and “unclassified” due to their heterogeneity from classical T1DM and T2DM phenotypes [44]. 

## 3. Clinical Manifestations

DN is classified as a “length-dependent” neuropathy, as it appears to begin at the distal nerve endings of the longest neurons in the lower limbs and spreads proximally [9]. The clinical manifestations for DN are diverse, yet they are typically categorized as “painful DN”, with positive symptoms and gain of function (e.g., pain, hyperalgesia, allodynia), and “insensate DN”, with negative symptoms and loss of function (e.g., numbness, dysesthesia), indicating predominantly small and large fiber loss, respectively. Additionally, up to 50% of people with signs of DN remain asymptomatic [4]. Small fiber loss can be detected through a lack of thermal differentiation and pinprick sensation, whereas large fiber loss is typically demonstrated by diminished or missing ankle reflexes, vibration perception and protective sensation. It should be noted that concurrent mixed small and large fiber loss is frequent, resulting in both positive and negative signs and symptoms [4]. Changes in motor function, such as weakness, atrophy, gait disorder and loss of coordination, are typically noticed later in the course of DN; however, evidence suggests that these issues exist at a subclinical level [45]. Sensorimotor dysfunction can lead to unsteadiness and increase the risk of falls by a factor of 20 compared to matched non-diabetics [46], and recurrent falls can lead to physical and psychosocial trauma [3,46]. 

In contrast to the sensorimotor aspects of DN, the psychosocial impact has received little attention [3]. In the last decade, the term “diabetes distress”, defined as a diabetes-related hidden emotional burden, has been coined [47]. The emotional burden of diabetes, however, is not hidden. Studies have shown that people with diabetes have up to a 20% and 10% higher prevalence of anxiety and depression, respectively, compared to people without diabetes [48,49]. A recent systematic review reported the prevalence of anxiety (7.8% to 60.4%), depression (13.6% to 50.6%) and coexistence of the two conditions (26.4% to 30.6%) in people with painful DN [50]. The large variation in estimates may be attributed to differences in definitions and assessments of mental health conditions. Sleep disorders, on the other hand, have a more defined and consistent prevalence (41.6% to 43.8%), indicating that they are a common and potentially debilitating comorbidity [50]. People with diabetes and mental health comorbidities have a greater risk of developing DN as well as other complications such as cardiovascular disease, metabolic syndrome, and sexual dysfunction [51,52,53]. In addition, they are less likely to adhere to self-monitoring, which can contribute to poor glycemic control and the development of diabetic foot ulcers (DFUs) [54,55]. 

There is a strong link between DN and DFUs. As many as 25% of people with diabetes will develop DFUs [56], with the majority being neuropathic or neuro-ischemic in origin [57,58,59]. DFUs are difficult to heal and are compounded in DN by the absence of foot sensation and pain, which results in patients unknowingly walking on active wounds, resulting in impaired healing [60]. DFUs are associated with a reduced QoL; a cross-sectional study (n = 310) found that patients with active DFUs scored significantly lower on EQ-5D compared to those with healed DFUs [61]. DFUs are also associated with high rates of mortality; a systematic review of 12 studies reported a five-year mortality rate of approximately 40% [62], which increases to 50% at two years after a major lower limb amputation [63]. In the UK, DFUs are estimated to cost the National Health Service (NHS) GBP 1 billion per year [6].

A rarer complication of DN is Charcot neuropathic osteoarthropathy (‘Charcot foot’), a condition that causes gradual bone and soft tissue destruction and deformity. Despite diabetes being the leading cause of Charcot foot in the northern hemisphere [60], the rates in people with diabetes are unknown; estimates place the incidence between 0.1–0.9% per year [64]. Patients with Charcot foot are typically younger than those with DFUs, and its early stages present with warmth and oedema around the foot secondary to inflammation of the bones, joints and soft tissue. This can easily be misdiagnosed as cellulitis or gout [65,66]. Later deformities, such as a collapsed midfoot arch, occur from chronic bone demineralization, fractures and joint dislocation [65]. It is also a major risk factor for mortality; a study comparing mortality data in patients with Charcot foot (n = 70) and normative population data discovered that the presence of Charcot foot is associated with a 14-year decrease in life expectancy [67]. Limited information is available on the overall costs of Charcot foot, most likely because the true prevalence is unknown. However, estimates from studies conducted in the United States (USA) suggest that costs range from $20,000 to $60,000 per patient [68,69]. 

## 4. Prevention Strategies

Current prevention strategies for DN focus on glycemic control, lifestyle modifications and footcare. Table 1 summarizes the main advantages and disadvantages of these strategies. 

### 4.1. Glycemic Control

Achieving glycemic control to prevent DN is recommended in several clinical guidelines [2,4,90,91]. As aforementioned, the DCCT/EDIC studies demonstrated that glycemic control is strongly linked to DN in people with T1DM. In the DCCT, intensive glucose monitoring reduced the incidence of DN by 69% at five years [36]. The UK Prospective Study (UKPDS) [92], a major trial exploring glycemic treatments in people with T2DM, has been argued to have found similar trends to the DCCT/EDIC findings [38,93]. Although overall microvascular complications were reduced by 25% after ten years of glycemic treatment, the reduction in DN alone was not statistically significant (16%, *p* = 0.033) [92,94]. The American Diabetes Association’s interpretation of the UKPDS findings is that glycemic control prevents retinopathy, nephropathy, and “possibly” neuropathy [94]. Furthermore, the ACCORD trial demonstrated that intensive glycemic management did not reduce the risk of DN in people with T2DM but did delay its onset [35].

These observed differences in the efficacy of glycemic control when comparing T1DM and T2DM have been further examined in a Cochrane systematic review and meta-analysis [70]. The authors identified 17 randomized controlled trials (RCTs) investigating enhanced glycemic control in the prevention of DN (seven conducted in people with T1DM, eight in people with T2DM and two in both). In people with T1DM, enhanced glycemic control significantly reduced the risk of DN (annualized risk difference −1.84%; *p* < 0.00001). The risk was also reduced in people with T2DM (annualized risk difference −0.58%; *p* = 0.06); however, this did not reach statistical significance [70]. This could be attributable to heterogeneity in conducting DN assessments across trials. Glycemic control in isolation may also be insufficient for people with T2DM because they are more likely to have additional cardiometabolic risk factors that go unaddressed [95]. Interestingly, a randomized parallel trial of conventional therapy versus intensive therapy targeting glycemic control and cardiometabolic risk factors via pharmacotherapies, diet, exercise and behavior modification in people with T2DM (n = 160) found that intensive therapy significantly reduced the risk of autonomic neuropathy (hazard ratio 0.37; *p* = 0.002) but not DN (hazard ratio 1.09; *p* = 0.66) at 8 years follow-up [96]. 

Glycemic control can now be assessed more readily with flash glucose monitors (FreeStyle Libre) and continuous glucose monitoring (CGM). A recent meta-analysis found that CGM tools significantly improve glycemic control. Fifteen RCTs (n = 2461) comparing the effects of CGM versus standard care (typically self-blood glucose monitoring) on glycemic control were identified, and pooled analysis revealed that CGM significantly increased time in target range while decreasing time above and below range, as well as glucose variability [97]. Clinical guidelines recommend tailoring glycemic targets to the individual. The American Diabetes Association has published separate glycemic target guidelines for children and adolescents, adults, pregnant adults and older adults, all of which promote individualized care [71,72,73,74]. In the UK, the National Institute for Health and Care Excellence (NICE) recommends shared decision making for glycemic targets using a patient decision aid that takes into account the challenges of achieving glycemic control, such as hypoglycemic episodes, side effects of anti-diabetic medications, and risks of treatment-induced neuropathy (“insulin neuritis”) and potentially other acute neuropathies [75,76,77,78,79]. These advances in glucose monitoring, which are also becoming more accessible to patients, could prove to be an effective strategy, especially in patients with T1DM, where the benefits in terms of DN are more evident. 

### 4.2. Lifestyle Modifications 

To reduce the risk of DN, prevent disease progression and minimize cardiometabolic risk factors, some clinical guidelines recommend lifestyle modifications such as regular exercise and a balanced diet [2,4,91]. The American Diabetes Association proposes these changes specifically for people with pre-diabetes, metabolic syndrome and T2DM [2,4]. Contrastingly, other neuropathic pain guidelines make no mention of lifestyle modifications [98,99], and the American Academy of Neurology has previously stated that exercise has no efficacy for painful DN [100], which could be due to these guidelines mainly considering symptomatic treatment of neuropathic pain, not prevention of disease progression. 

Exercise has a moderate level of evidence for the prevention and treatment of DN. A recent meta-analysis (13 RCTs, 592 participants) found that exercise interventions may improve balance, peripheral nerve conduction velocity and glycemic control in patients with DN, with a combined endurance and sensorimotor training program being the most beneficial [80]. For example, this could entail moderate-intensity cycling and progressive balance exercises on uneven surfaces a few times a week for 12 weeks. The meta-analysis included a randomized controlled trial (RCT) (n = 78) conducted by Balducci et al. [83] with a four-year exercise intervention period, the longest duration studied in people with DN. Participants randomized to a supervised exercise program of 4 h per week had significantly different nerve conductivity at the common peroneal and sural nerves than those randomized to no program and complied excellently attending > 90% of the sessions. Furthermore, the number of participants who developed motor and sensory neuropathy during the four-year study period was significantly higher in the control group than the intervention group, suggesting supervised exercise may alter the natural course of DN [83]. A detailed overview of outcomes from other supervised exercise programs can be found in Table 1. Implementing supervised exercise programs outside of research settings may be difficult due to patient compliance and a lack of funding, staff and infrastructure to supervise exercise programs in healthcare systems.

In people with pre-diabetes, the level of evidence for exercise is low and requires further investigation. Only one completed study has been identified in people with prediabetes, which found that after one year of individualized exercise (150 min per week) and dietary advice, intraepidermal nerve fiber density increased significantly, as measured by nerve biopsy at the lower limb. This increase was also associated with improved electrophysiological and pain outcomes, validating intraepidermal nerve fiber density as a surrogate measure for small fiber neuropathy. The study, however, was not powered or designed to investigate efficacy [82]. A powered, multi-center, international RCT on people with prediabetes is ongoing; the ePREDICE trial (clinicaltrials.gov identifier NCT03222765) aims to compare the effects of early glycemic control with antidiabetic medications plus lifestyle modifications (exercise, diet, behavior, motivation) versus lifestyle modifications alone on DN and will provide valuable data on these prevention strategies [101]. While the effects of exercise on DN in people with pre-diabetes may be inconclusive due to a scarcity in data, this only highlights the need for further research in this area. Nevertheless, diet, weight management and exercise interventions should be encouraged for people with pre-diabetes as per T2DM guidelines [102,103]. In addition, there is moderate evidence to suggest that exercise may reduce the occurrence of DFUs. A recent systematic review (six studies, 418 participants) found that ulcer incidence was lower in exercise intervention groups compared with control groups (0.02 vs. 0.12 per year, respectively) [81]. 

There is a paucity of studies investigating diet alone in the context of DN because most studies include diet as part of a multifactorial lifestyle intervention. Nonetheless, the American Diabetes Association advises to reduce calorie and processed food intake while increasing polyunsaturated fats and antioxidant rich foods in order to minimize risk factors and improve outcomes [2]. Nourishment may be especially important for people with diabetes following bariatric surgery, because increased rates of DN have been reported in this group, potentially due to nutritional deficiencies [104]. Other studies, however, have suggested that bariatric surgery may improve DN [105,106]. 

With physical activity and improved diet being cornerstones of prevention guidelines, psychological and behavioral change interventions may help people with diabetes improve compliance with such lifestyle modifications. Currently, there are no guidelines that recommend counselling for the prevention of DN, despite its considerable psychosocial burden (including pain). A previous systematic review of 25 RCTs found improved long-term glycemic control in people with T2DM following psychological therapies and commented positively on less “psychological distress”, though improvements in body weight and blood glucose levels were not found [84]. A more recent systematic review and meta-analysis of 13 RCTs investigating behavior change techniques targeting both diet and physical activity through calorie restriction and increased aerobic activity in people with T2DM found clinically important improvements in glycemic control in the short term but not the long term, and a reduction in body weight across all timepoints [85]. While available studies do not typically assess progression to DN, psychological support and diabetes-related counselling should play a more dominant role in holistic management of people with diabetes.

### 4.3. Footcare

Clinical guidelines in the USA recommend that people with T1DM have a foot assessment five years after diagnosis and then annually thereafter, and that people with T2DM have foot assessments both at diagnosis and on an annual basis [2,4,90]. Alternatively, in the UK, NICE recommends that all people with diabetes have a foot assessment upon diagnosis and annually, regardless of whether they have T1DM or T2DM [107]. These checks are an important opportunity to assess the risk of ulceration, modify abnormal risk factors and deliver patient education. 

The ‘Putting Feet First’ framework exists to outline the minimum diabetic footcare required in the UK. It emphasizes the importance of a multidisciplinary approach involving diabetology, podiatry, vascular surgery, orthopedics and other specialties, as well as a pathway to ensure timely footcare and urgent referral [108]. For example, transfer of care to dedicated clinics that specialize in strategies such as off-loading, debridement, managing infection and restoring arterial flow, in order to avoid amputation, are essential and provide optimal limb salvage treatment [66,87]. However, a recent national audit in the UK found that these services are currently fragmented and confusing, leaving patients in the community without the necessary treatment [88]. This is a major concern, because it has been reported that with the appropriate treatment, complications such as Charcot foot are completely preventable [66]. 

The level of evidence for footcare in preventing further foot complications is low. A systematic review identified 19 studies that evaluated the impact of multidisciplinary care on diabetic foot outcomes. Although amputation severity, mortality rates and length of hospital stay were reduced, the studies reviewed were of low-quality [86]. Furthermore, a previous Cochrane systematic review of 12 RCTs found insufficient high-quality evidence to determine whether the use of educational strategies reduces the incidence of DFUs and amputations [89]. 

## 5. Diagnostic Evaluation

Several guidelines include recommendations for diagnostic evaluation of DN. They emphasize the importance of accurate diagnosis, which excludes other causes of peripheral neuropathy, as well as regular evaluation so that DN is detected early, allowing for advanced implementation of strategies to prevent disease progression [2,4,90,91]. A discussion of the breadth of diagnostic evaluation tools currently available and under investigation, including their specificity and sensitivity, is beyond the scope of this article (though we refer the reader to relevant review articles [109,110,111]); instead, a summary and discussion of major challenges is provided.

There are numerous diagnostic and screening tools for DN available; though many focus on symptomatology and may overlook people with early DN. The Michigan Neuropathy Screening Instrument, for example, is a validated screening tool for DN that includes a patient questionnaire and separate clinical examination of the feet [112]. At validation, a patient questionnaire score of ≥7 and a clinical examination score of ≥2.5 were considered abnormal [112]. Since then, the threshold for the patient questionnaire score has been called into question, with subsequent evidence suggesting that a score of ≥4 may be more sensitive [113], and another recent study defining a score of ≥2, neglecting the clinical examination score altogether [114]. Although these developments may capture people with earlier disease, further research into their validity is needed, and the reliance on clinical symptoms remains. The Toronto Clinical Severity Score is another widely used tool that assesses symptoms, reflexes and sensation in people with suspected DN. It also has the added benefit of stratifying patients by severity, which is associated with neurophysiology measures [115]. However, it also relies heavily on the presence of clinical symptoms (worth a maximum of six out of 19 points) to yield a positive result, which means it may not identify the approximately 50% of people with DN who are asymptomatic and those with mild disease, groups that may benefit the most from prevention strategies [4]. Alternatively, the Neuropathy Impairment Score in the Lower Limbs (NIS-LL) [116,117] does not assess symptoms but places an emphasis on weakness, reflex and sensation outcomes [118]. However, it has been argued that this focus on motor function captures people with predominantly large fiber dysfunction and not small fiber dysfunction, the latter being an earlier pathophysiological finding [119].

The gold standard for diagnosing DN is nerve conduction studies, though there are inconsistencies in how these are undertaken and interpreted. Dyck et al. [120] developed a classification system that defined DN grades based on the percentile of nerve conduction abnormalities, signs and symptoms [120]. The disadvantage of this system is that the inter-operator variability of nerve conduction studies is high [121], and population percentiles are not widely available, so the neurophysiologist must rely somewhat on clinical judgement to determine grades. An alternative approach is the Baba classification system [122] that bases DN severity on clearly defined sural and tibial nerve conduction parameters. However, this system may overlook important longitudinal changes in nerve conduction because it does not take into account common peroneal and median nerve conductivity, which have been shown to improve significantly following glucose monitoring [70]. In addition, as with all nerve conduction studies, any changes in small fiber function are not captured, which is critical given that these fibers are targeted first.

The gold standard for assessing small fiber function is intraepidermal nerve fiber density, but this requires an expensive, invasive skin biopsy. The procedure can be unsettling for patients and results in a small wound, raising safety concerns due to an increased risk of foot ulceration in this patient group. As a result, non-invasive alternatives have emerged such as Quantitative Sensory Testing (QST), a measure of sensory function that includes sensation involving small fibers. The German Research Network on Neuropathic Pain has developed a valid, comprehensive, standardized protocol for QST [123], which includes measures of temperature, pain and vibration thresholds. This reduces the risk of inter-operator variability, but intensive training is still required to maintain low intra-operator variability. This training may be considered expensive, and additional costs for licenses and access to normative data apply.

Several novel diagnostic evaluation tools are under investigation. The most notable is corneal confocal microscopy, a noninvasive imaging technique that assesses small (C) fiber damage in the cornea, the body’s most densely innervated tissue [124]. A systematic review and meta-analysis of 13 studies (1680 participants) found that people with DN have significantly lower corneal nerve fiber density, length and branch density compared to healthy controls, implying that corneal confocal microscopy may be a useful tool in assessing early nerve damage [125]. Furthermore, new evidence suggests these corneal nerve changes are strongly linked to neuropathic pain [126]. According to a recent review by Petropoulos et al. [124], the last 20 years of research into corneal confocal microscopy has yielded sufficient evidence to classify corneal nerve loss as a biomarker for DN because it can predict incidence and progression, but this is yet to be recognized by regulators [124]. Corneal confocal microscopy may provide an easy and accurate test for detecting early DN, and further high-quality research should be undertaken to strengthen its evidence.

## 6. Management Strategies

As the management strategies for people with insensate or painless DN are limited, current guidelines focus on strategies for painful DN. These include pharmacotherapies such as anticonvulsants, serotonin and norepinephrine reuptake inhibitors (SNRIs), tricyclic antidepressants (TCAs), opioids, topical analgesics and intravenous (IV) medications. Table 2 summarizes the main advantages and disadvantages of these strategies. 

### 6.1. Anticonvulsants 

Pregabalin and gabapentin are recommended first- or second-line treatments for painful DN [2,4,91,98,99,100]. In the USA, pregabalin is Food and Drug Administration (FDA) approved for this indication, whereas gabapentin is only licensed to treat postherpetic neuralgia and is therefore being used to treat a non-indication [129]. Both are licensed more broadly in the UK to treat peripheral neuropathic pain [130,132]; however, recently, these medicines have been more tightly regulated due to incidents of misuse [131].

The level of evidence for these medicines as a treatment for painful DN is moderate. A recent Cochrane systematic review found that pregabalin 300 mg significantly reduced pain intensity by at least 30% (risk ratio 1.1—eight RCTs, 2320 participants) and 50% (risk ratio 1.3—11 RCTs, 2931 participants) versus placebo [127]. The results were comparable in gabapentin; there was a moderate (seven RCTs, 1439 participants) and substantial (six RCTs, 1331 participants) benefit with gabapentin 1200 mg or greater versus placebo [128]. Studies on other anticonvulsants such as carbamazepine, valproic acid and lamotrigine and phenytoin have been undertaken; however, there is limited evidence for their use [162,163,164,165].

The exact mechanism by which pregabalin and gabapentin act are unknown. They bind to the α2δ subunit of calcium channels, which appears to result in analgesic effects; however, they are associated with tachyphylaxis. Typical side effects for both include drowsiness, dizziness, headache, diarrhea and nausea [130,132]. In 2021, following a review of international safety data, the Medicines and Healthcare products Regulatory Agency (MHRA) in the UK published a further safety update for pregabalin, stating that it is linked to infrequent reports of severe respiratory depression and that dosing adjustments should be considered for those at higher risk, such as people over the age of 65, with respiratory or neurological disease or renal impairment [133]. 

### 6.2. Serotonin and Norepinephrine Reuptake Inhibitors (SNRIs)

Duloxetine is also recommended as a first- or second-line treatment for painful DN [2,4,91,98,99,100]. It is approved in the USA and UK specifically for DN, but not for other neuropathic pains [129,143]. As an SNRI, duloxetine inhibits descending pain pathways and modestly inhibits dopamine reuptake. Typical side effects are similar to those of anticonvulsants, though sexual dysfunction and sleep problems may be more noticeable [4,143].

The level of evidence for duloxetine is also moderate; a previous Cochrane systematic review (eight trials, 2728 participants) found that 60 to 120 mg is efficacious in treating painful DN, while lower doses are not [134]. Since the Cochrane review, there has been a large-scale (n = 405), multi-center, double-blind RCT that demonstrated 60 mg of duloxetine daily for 12 weeks significantly reduced DN pain versus placebo [166]. Comparative trials have produced mixed results [167], with some demonstrating that duloxetine has a similar effect to gabapentin, pregabalin and amitriptyline (a tricyclic antidepressant) [136,137,138,139]. Other trials, however, have shown duloxetine to be more effective than pregabalin [140,141,167]. 

Venlafaxine, an SNRI and selective serotonin reuptake inhibitor (SSRI), is recommended as a first- or second-line treatment for painful DN by the European Federation of Neurological Societies Task Force and the American Academy of Neurology [99,100], but it is not licensed for this use. Other clinical guidelines support its efficacy, but make no particular recommendations [4,91], while NICE advises against using this medication in a non-specialist setting [98]. It is worth noting that the American Diabetes Association’s most recent monograph on the treatment of painful DN does not mention venlafaxine [2].

The level of evidence for venlafaxine is low. A previous Cochrane systematic review (six RCTs, 460 participants) found little convincing evidence to support the use of venlafaxine in the treatment of painful DN. Although doses ranging from 150 to 225 mg showed some benefit, these studies were compromised by a large placebo effect [142]. There has not been a comparative trial of venlafaxine and duloxetine in DN; however, in chemotherapy-induced peripheral neuropathy, duloxetine was superior [168].

### 6.3. Tricyclic Antidepressants (TCAs)

Alternatively, amitriptyline is recommended as a first- or second-line treatment for painful DN [4,91,98,99,100]. It is the only TCA licensed in the UK for the treatment of neuropathic pain [145], though it is not licensed for this use in the USA. It is primarily used to treat major depressive disorder.

The level of evidence for amitriptyline is low. Although several RCTs have indicated a reduction in DN pain with amitriptyline [136,169,170,171,172,173], a previous Cochrane systematic review (five studies, 654 participants) concluded that the data presented are likely biased, owing to the small sample sizes. The authors did, however, underline that this conclusion should be balanced against the fact that amitriptyline has been beneficial to thousands of people [144].

The exact mechanism by which amitriptyline acts is unknown, though it is known that the antidepressant and analgesic actions are distinct. It inhibits noradrenaline and serotonin reuptake at nerve terminals, as well as sodium, potassium and N-methyl-D-aspartate (NMDA) channels in the central nervous system, which are known to be involved in neuropathic pain [174]. A starting daily dose of 10 to 25 mg is recommended, which can be gradually increased to 75 mg per day. Typical side effects include sleep disorders, dry mouth, constipation, sexual dysfunction, arrythmias, headaches and postural hypotension [145], the latter of which can be especially problematic in elderly patients and should be closely monitored [174].

Desipramine and nortriptyline have also been investigated for their efficacy in the treatment of painful DN. Multiple RCTs have shown that desipramine significantly reduced symptoms of painful DN [173,175,176], and may have a similar treatment effect to amitriptyline [176]. Furthermore, a small (n = 16), double-blind RCT found that nortriptyline significantly reduced pain scores in people with DN [177]. Despite this, previous Cochrane systematic reviews have concluded there is no evidence to support their use in the treatment of neuropathic pain, owing to studies being methodologically flawed and subject to major bias [178,179]. This position is also echoed by the American Academy of Neurology [100]; however, the American Diabetes Association has suggested these agents are preferable to amitriptyline for elderly patients and people with certain comorbidities [4]. There have been no recent trials on desipramine and nortriptyline in DN, although a new trial in cryptogenic sensory polyneuropathy demonstrated nortriptyline was successful in reducing neuropathic pain [180].

### 6.4. Opioids

Some clinical guidelines recommend considering opioids for the treatment of painful DN [99,100], or as a part of combination therapy if pain cannot be controlled [91]. However, opioids present a major challenge in terms of misuse and abuse [152]. Given these challenges and other risks, the American Diabetes Association does not recommend opioids as a treatment for painful DN [2], and according to NICE, only tramadol should be used as an acute salvage treatment [98].

Tramadol is licensed in the USA and UK for moderate to severe pain rather than specifically neuropathic pain [129,150]. It binds centrally to the δ, κ and μ, receptors, with the latter having the greatest affinity [181]. In addition, it inhibits the reuptake of noradrenaline and serotonin and therefore should not be taken in combination with SNRIs/SSRIs [99]. Compared to other opioids, tramadol may have a decreased risk for abuse [151].

Overall, the level of evidence for tramadol is low. Two major RCTs have shown that tramadol significantly reduces pain in people with DN versus placebo [148,149]. A subsequent RCT in people with polyneuropathy, including diabetics, further demonstrated the analgesic efficacy of tramadol [182]. However, a recent Cochrane systematic review concluded that there is limited information on the use of tramadol in neuropathic pain and trials have been small and likely biased [146].

Tapentadol is licensed specifically for neuropathic pain in the USA but not in the UK [129]. The analgesic action is similar to tramadol, except tapentadol has a higher affinity for the μ receptor [167]. Typical side effects of tapentadol and tramadol include dizziness, drowsiness, headache, nausea and constipation [150]. Currently, the level of evidence for tapentadol is low. Although several trials have shown it to be beneficial in the treatment of painful DN [183,184,185], further high-quality RCTs are needed [147].

Other opioids, such as methadone and oxycodone have insufficient evidence to support their use in the treatment of neuropathic pain [186,187]. Further RCTs are required to assess efficacy, safety and impact on QoL [188].

### 6.5. Topical Analgesics

Some clinical guidelines recommend considering topical analgesics for painful DN [98,99,100], especially in people with localized pain who are unable to tolerate oral medications [98]. Topical capsaicin has recently been approved by the FDA for painful DN [2], but it is not licensed in the UK for this indication. It is hypothesized to reduce pain by altering membrane potential and neurotrophic signaling at the nerve fiber [189].

The level of evidence for topical capsaicin ranges from moderate to low based on the strength applied. An RCT investigating 0.025% capsaicin gel demonstrated no significant effect in reducing pain versus placebo in people with painful DN [190]. Meanwhile, a previous meta-analysis (six RCTs, 656 patients) determined that 0.075% capsaicin significantly reduces neuropathic pain versus placebo [156]. Since the meta-analysis, an adequately powered double-blind RCT has demonstrated no significant difference between 0.075% capsaicin and placebo [157].

A recent meta-analysis (25 RCTs) of 8% capsaicin in the treatment of painful DN found that the patch is more efficacious in achieving ≥ 30% pain reduction versus placebo. Furthermore, it may be more beneficial than anticonvulsants and have a similar efficacy profile to duloxetine [154]. A recent post hoc analysis of the multi-center, open-label trial PACE found that, while pain reduction can be achieved with a single application, some patients may require two to three applications before achieving a treatment response [155]. There are concerns that topical capsaicin can cause small nerve fiber injury and as a result disturbed nociceptive signaling [158]. In people with affected nociceptors due to capsaicin, topical clonidine, an α2 adrenergic receptor agonist, has been found to be superior [191]. Despite these concerns, topical capsaicin may be more tolerable than other oral pain medications [154].

The level of evidence for other topical analgesics, such as topical lidocaine and topical ketamine, are very low as there are no high-quality RCTs demonstrating that these agents have efficacy for DN [192,193,194].

### 6.6. Intravenous (IV) Medications

Clinical guidelines do not currently recommend IV medications for painful DN, though there is some low-quality evidence to suggest these may be beneficial and warrant further investigation. A recent systematic review (26 studies) found that IV lidocaine is effective in reducing neuropathic pain in the short term [159]. Since the systematic review, an RCT in 34 people with refractory neuropathic pain found no additional analgesic effect with IV lidocaine versus the control infusion. In this study, typical side effects reported post infusion were somnolence, dizziness, nausea, and abdominal pain [160].

IV ketamine produces analgesia and anti-hyperalgesia when administered at sub-anesthetic dosages [152]. A recent systematic review identified 13 studies investigating IV ketamine for neuropathic pain, and all demonstrated an analgesic effect [161]. Typical side effects of ketamine include dizziness, drowsiness, lack of appetite, nausea and vomiting [160].

## 7. Novel Therapies

Neuromodulation devices such as spinal cord stimulation (SCS), frequency-modulated electromagnetic neural stimulation (FREMS), transcutaneous electrical nerve stimulation (TENS) and neuromuscular electrical stimulation (NMES) are among the novel therapies under investigation. In addition, nutraceuticals such as α-lipoic acid (ALA), vitamin B12 and acetyl-L-carnitine (ALC) are being increasingly investigated for their safety and efficacy in DN as per recommendations from the American Academy of Neurology [100]. The American Diabetes Association has recognized this, recently including a discussion of nutraceuticals in their monograph on the treatment of painful DN [2], despite previously holding that they lacked evidence for their use in diabetes [195]. Nutraceuticals are an attractive treatment prospect for DN because they are widely available, inexpensive and generally regarded as “safe”, but concerns remain due to their lack of regulations including standardization in manufacturing and quality [195,196]. In addition, the current evidence for their safety remains low to very low due to a lack of high-quality studies [197,198].

### 7.1. Neuromodulation Devices

#### 7.1.1. Spinal Cord Stimulation (SCS)

SCS is recommended by some clinical guidelines for severe painful DN [2,91]. There are three types of SCS therapies available which administer different electrical impulses: conventional (tonic pulse, frequency between 40 and 80 Hz), high-frequency (tonic pulse, frequency between 1 kHz and 10 kHz) and burst stimulation (intermittent stimulation, varying parameters). The exact mechanism through which SCS reduces pain is unknown [199]. SCS is approved in the UK for refractory neuropathic pain and the FDA approved a 10 kHz SCS system for refractory painful DN in the USA in 2021 [200,201].

The level of evidence for SCS is low. A recent systematic review identified only two multi-center RCTs investigating SCS for painful DN [202]. Although both RCTs were significant in reducing pain [203,204], the design of these were low-quality and likely subject to high risks of bias [202]. A recent Cochrane systematic review (15 RCTs, 908 participants) of the efficacy of SCS in people with chronic pain (not exclusively DN) found low-quality evidence, and that any treatment effect disappeared once trials were sham controlled. Furthermore, it highlighted the serious adverse effects linked to this therapy such as infection, lead failure/displacement and a need for further surgical procedures [199].

Recently, dorsal root ganglion stimulation has also been proposed as an alternative nonpharmacological treatment for painful DN [205,206], but searches identified no RCTs investigating its efficacy.

#### 7.1.2. Frequency-Modulated Electromagnetic Neural Stimulation (FREMS)

Clinical guidelines do not recommend FREMS as a treatment for DN as the level of evidence is very low. A preliminary RCT (n = 31) demonstrated that two rounds of ten FREMS sessions significantly reduced pain levels and vibration thresholds and improved sensory tactile perception and motor nerve conductivity, whereas no significant changes were seen with the placebo device [207]. A subsequent multi-center, double-bind RCT (n = 110) primarily assessing if FREMS improved peripheral nerve conductivity found no significant difference between the intervention and controls groups. Additionally, although pain decreased during FREMS administration, this did not last after stimulation [208]. In addition, the recently published FREMSTOP study (n = 25) found no statistically significant differences in pain levels from baseline and 12 weeks following ten FREMS sessions [209].

#### 7.1.3. Transcutaneous Electrical Nerve Stimulation (TENS)

Although TENS is a nonpharmacological treatment option for chronic pain, no clinical guidelines recommend its use in painful DN. The level of evidence for TENS is low; a meta-analysis found that TENS significantly reduces pain scores by −0.44 (95% CI −0.79 to −0.09), but the studies included were of low methodological quality [210]. There have been small studies in people with DN which have indicated that TENS may improve vibration perception thresholds, balance and gait parameters versus sham devices [211,212].

A modality very similar to TENS is electrical stimulation via percutaneous needles known as PENS (percutaneous electrical nerve stimulation). Despite the American Academy of Neurology previously recommending PENS as a treatment for painful DN [100], the level of evidence is very low. There has been one RCT (n = 50) in adults with T2DM and DN, which showed that PENS significantly improved pain, physical activity, and sleep scores versus sham needles [213]. Further research with well-designed RCTs is required for both TENS and PENS.

#### 7.1.4. Neuromuscular Electrical Stimulation (NMES)

NMES has the potential to be a nonpharmacological adjuvant treatment for DN. It differs from other peripheral nerve stimulation modalities, such as TENS, in that it is applied at a sufficient intensity to depolarize neurons and evoke muscle contraction, simulating exercise. A double-blind RCT investigating NMES in DN is currently ongoing (clinicaltrials.gov identifier NCT03767478).

### 7.2. Nutraceuticals

#### 7.2.1. α-Lipoic Acid (ALA)

The most promising nutraceutical in the treatment of DN is ALA, though its potential has only been recognized in some clinical guidelines [2,91]. ALA is a naturally occurring antioxidant that may act as a treatment for DN by reducing oxidative stress, which affects both nerves and microvessels via multiple metabolic mechanisms. It is licensed to treat DN in some countries, but not in the US or the UK [2].

The level of evidence for ALA is low. A previous meta-analysis (15 RCTs) found motor and sensory nerve conduction velocities to be significantly improved in intervention groups administering 300 to 600 mg per day IV ALA compared to control groups. However, many of the included studies were low-quality and subject to a high risk of bias [197]. Oral ALA has also been investigated; the double-blind RCT SYDNEY II (n = 181) conducted across Russia and Israel demonstrated that 5 weeks of 600 mg per day ALA significantly improved neuropathy symptoms versus placebo, and is superior to doses of 1200 mg and 1800 mg [214]. However, the NATHAN 1 trial found that 600 mg per day ALA across four years had no significant effect on the composite primary endpoints of NIS-LL score and a battery of neurophysiology tests [215]. Despite significant improvements in NIS-LL scores from baseline, the FDA will not accept evidence for approval if the primary endpoint is not achieved [7,215].

#### 7.2.2. Vitamin B12

There are important links between vitamin B12 deficiency, peripheral neuropathy and the treatment of diabetes. Firstly, vitamin B12 deficiency is an independent cause of peripheral neuropathy, and its exclusion is required for a diagnosis of DN [4]. Second, metformin, a T2DM treatment, can lead to vitamin B12 deficiency. A recent meta-analysis (31 studies) found that people taking metformin had a significantly higher risk of vitamin B12 deficiency (risk ratio 2.09; *p* < 0.0001) and lower vitamin B12 levels (mean differences −63.70 pM; *p* < 0.00001) compared to people taking other diabetes treatments [216]. Thus, the American Diabetes Association recommends regular vitamin B12 testing for people being treated with metformin, particularly those with anemia or peripheral neuropathy [217]. These recommendations are also followed in the UK, with a 2022 MHRA report indicating that the incidence of vitamin B12 deficiency in people taking metformin is much higher than expected [218]. Vitamin B12 is hypothesized to treat DN by promoting myelin production and repair and reducing neuropathic pain, though the exact analgesic mechanism is unknown [219,220,221].

The level of evidence for vitamin B12 is low. Vitamin B12 can be administered by injection or orally, but the latter has been investigated in most studies. A notable double-blind RCT that included people with DN and autonomic neuropathy who had been taking metformin for at least four years investigated the effects of oral vitamin B12 (1000 μg/day) versus placebo after one year. Compared to baseline, participants in the intervention group (n = 44) had significantly improved neuropathy symptoms, pain outcomes, vibration perception threshold, QoL, sural nerve conduction velocity and action potential amplitude and skin conductance in their feet, while cardiovascular autonomic reflex texts and neuropathy clinical examination scores were non-significantly improved. In addition, no participants reported any suspected or related adverse effects [222]. This study is encouraging, but more high-quality research is needed to determine its efficacy and safety in DN.

Presently, vitamin B12 is only indicated for people who have a deficiency, so it is not suitable for everyone with DN [2,223]. There is a lack of data on its use in T1DM, as most studies have focused on people with T2DM who are being treated with metformin. There has been a multi-center double-blind RCT, undertaken by Li et al. [224], which enrolled people with T1DM and T2DM to comparatively investigate vitamin B12 (0.5 mg/three times a day) and ALC (500 mg/three times a day). At 24 weeks, both groups had significantly reduced neuropathy symptoms and disability, as well as improved nerve conductivity, though the vitamin B12 group did not improve in the sural and common peroneal nerve action potential amplitudes. Overall, the agents were well tolerated and participants in both groups experienced similar adverse events, which included gastrointestinal issues, abdominal discomfort, hiccups and nausea. The authors did not report any differences between diabetes subtypes, which may have been insightful and is encouraged in future research [224]. Other side effects reported with vitamin B12 in the general population include acne and sensitization, but these are very rare [223].

#### 7.2.3. Acetyl-L-Carnitine (ALC)

ALC has been proposed as a treatment for DN because of its role in nerve glucose and lipid metabolism. It promotes long-chain fatty acid transport, which is especially important in hyperglycemic and hyperlipidemic conditions to keep mitochondrial function efficient [198]. The level of evidence is low to very low. A recent Cochrane systematic review identified four studies (907 participants) investigating ALC in DN at varying doses of 2000 mg/day, 15,000 mg/day and 3000 mg/day. The review focused on pain outcomes at six and 12 months and concluded that there is low-quality evidence that ALC reduces pain in DN compared to placebo. Other outcomes, such as sensation and symptoms, had very low-quality evidence, and there was insufficient evidence to determine safety [198]. More high-quality research is needed, with outcomes such as nerve conductivity included.

## 8. Conclusions

**Figure 1:**
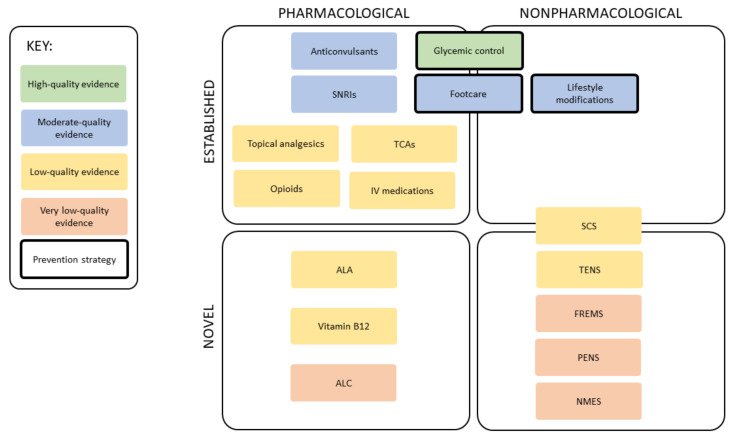
**Overview of prevention and management strategies for diabetic neuropathy.** ALA, α-lipoic acid; ALC, acetyl-l-carnitine; FREMS, frequency-modulated electromagnetic neural stimulation; IV, intravenous; NMES, neuromuscular electrical stimulation; PENS, percutaneous electrical nerve stimulation; SCS, spinal cord stimulation; SNRIs, serotonin and norepinephrine reuptake inhibitors; TCAs, tricyclic antidepressants; TENS, transcutaneous electrical nerve stimulation.

The rise in DN and its serious consequences for patients is both concerning and costly. More than 50% of people with diabetes will develop DN, causing symptoms ranging from numbness to unsteadiness and pain, as well as complications such as foot ulceration and Charcot foot. More recently, the psychosocial consequences of DN and their contribution to high levels of morbidity have also been recognized. Guidelines currently recommend two types of strategies: prevention, which aims to address DN before symptoms develop, or to prevent the progression of DN; and management, which investigates and treats symptoms patients already have while often failing to address the underlying cause. These established and novel strategies consist of pharmacological and nonpharmacological interventions (Figure 1).

The focus of DN prevention is glycemic control, lifestyle modification and footcare. Glycemic control can be achieved by a variety of different interventions such as insulin, antidiabetic medications, lifestyle modifications, pancreas transplant and bariatric surgery. In people with T1DM, glycemic control is an effective disease-modifying strategy; however, in people with T2DM, other cardiometabolic risk factors may also need to be targeted. Lifestyle modifications, such as supervised exercise programs, may improve DN outcomes and reduce the risk of DN and DFUs. Several different supervised exercise programs have been developed (i.e., endurance training, sensorimotor training, balance training, gait training, whole-body vibration, resistance training, physiotherapy and rehabilitation); each are beneficial, but they are difficult to implement in underfunded healthcare systems. A combination of endurance and sensorimotor training has been proposed as the most effective program; however, these types of programs frequently have low compliance. Personalized programs tailored to the individual may be more appealing. For example, balance training has been shown to be especially effective in treating psychosocial problems. Furthermore, offering diabetes and diet counselling may facilitate compliance with exercise programs, as well as assisting with glycemic control and weight management. To prevent further foot complications, multidisciplinary and self-led footcare through education are recommended, with multidisciplinary care including surgical and infection expertise the best approach for limb salvage; however, these services are currently underperforming, leaving patients confused and often being cared for in the community.

Screening and early diagnosis are critical for ensuring advanced implementation of strategies that prevent disease progression. There are numerous screening and diagnostic evaluation tools available, but many do not detect people with early DN because they are focused on symptoms or large fiber function. This is even true for nerve conduction studies, which are the current gold standard for diagnosing DN but are unable to assess small fibers that are targeted before large fibers. Although techniques for assessing small fiber function are available, they also have limitations. There are safety concerns around intraepidermal nerve fiber density, which is considered the gold standard for assessing small fibers, as it requires a skin biopsy leaving a wound in patients who are already at high risk of ulceration. Alternatively, QST is a noninvasive diagnostic modality including the assessment of small fiber function, but it is time consuming, expensive and requires training. Novel techniques, such as corneal confocal microscopy are being investigated and may provide an easy and accurate test for detecting early DN, but further high-quality research needs to be undertaken to strengthen its evidence for regulator review. There are few management strategies for people with insensate or painless DN. For people with painful DN, anticonvulsants, SNRIs and TCAs are recommended first- and second-line pharmacotherapies. These pharmacotherapies have moderate- to low-quality evidence and are associated with a variety of side effects. In some cases, opioids are recommended as third-line pharmacotherapies. New guidance, however, advises against their use entirely due to significant risks. The level of evidence for topical analgesics is moderate to low, but recent studies of 8% topical capsaicin show efficacy for painful DN. IV medications are not currently recommended and are limited to use in refractory cases and warrant further study.

Neuromodulation devices are novel, nonpharmacological therapies for DN that have the potential to improve pain outcomes and nerve conductivity. SCS has been recently approved for severe painful DN despite a level of low-quality evidence and its associated procedural risks. Other noninvasive neuromodulation devices such as TENS and NMES are being investigated, but there is a lack of data to determine their efficacy. Other innovative disease-modifying nutraceuticals have been explored, such as ALA, vitamin B12 and ALC, but none have received sufficient evidence for approval in the USA or UK and there are increasing concerns with their safety due to their lack of regulations.

## Figures and Tables

**Table 1:** **Advantages and disadvantages of prevention strategies for diabetic neuropathy.** DFUs, diabetic foot ulcers; DN, diabetic neuropathy; QoL, quality of life; T1DM, type 1 diabetes; T2DM, type 2 diabetes.

Prevention Strategy	Indication(s)	Intervention Types	Level of Evidence	Advantages	Disadvantages
Glycemic control	To reduce the risk of DN	Pharmacological: - Insulin - Antidiabetic medicines Nonpharmacological: - Lifestyle modifications - Pancreas transplant - Bariatric surgery	High-quality—all intervention types that enhance glycemic control for at least 12 months [70]	- Glycemic control reduces the risk of DN in T1DM (significant) and in T2DM (not significant) [70] - Glycemic control can be readily assessed with flash glucose monitors (FreeStyle Libre) and continuous glucose monitoring - Guidelines recommend that individualized glycemic targets are based on shared decision making [71,72,73,74,75,76]	- Enhanced glycemic control does not significantly reduce the risk of DN in T2DM [70] - Risk associated e.g., hypoglycemic episodes, side effects of anti-diabetic medications, treatment-induced neuropathy and potentially other acute neuropathies [77,78,79]
Lifestyle modifications	To reduce the risk of DN, to prevent progression of DN, to reduce cardiometabolic factors	Nonpharmacological: - Supervised exercise programs e.g., ∘ endurance training ∘ sensorimotor training ∘ combined endurance and strength training ∘ resistance training ∘ balance training ∘ combined balance and gait training/whole-body vibration/resistance training ∘ whole-body vibration ∘ physiotherapy/rehabilitation - Diet - Counselling	Moderate-quality—supervised exercise programs for DN and DFUs in people with diabetes [80,81] Low-quality—supervised exercise programs for DN in people with prediabetes [82], diet and counselling for DN in people with diabetes	- Endurance training may significantly reduce the risk of DN [83] - Supervised exercise programs may improve DN outcomes [80] ∘ endurance training may reduce neuropathic pain and may improve nerve conduction, symptoms, vibration perception threshold, blood glucose levels, daily function, arterial blood flow, QoL and relationships ∘ sensorimotor training may improve balance and mobility ∘ combined endurance and strength training may improve small fiber function and mobility ∘ balance training may reduce pain, tingling, anxiety, depression, concerns about falling, blood inflammatory markers and may improve QoL, mobility, trunk strength, function and blood glucose levels ∘ balance training combined with either gait training, whole-body vibration and resistance training may improve mobility, balance, vibration perception and gait and may reduce concerns about falling ∘ whole body vibration may improve mobility, balance, posture, blood glucose levels and lower limb strength ∘ physiotherapy/rehabilitation may improve mobility, balance and stability and may reduce fall risk - Supervised exercise programs can be personalized - Supervised exercise programs may reduce the risk of DFUs [81] - Diabetes and diet counselling may improve glycemic control and promote weight loss [84,85] - Counselling may also facilitate compliance with exercise programs - Lifestyle modifications provide a holistic approach	- The effects of resistance training on DN outcomes are inconclusive [80] - Patient compliance with supervised exercise programs is often low - There is a lack of infrastructure and resources to provide supervised exercise regimens in public healthcare systems - Long-term behavior change is challenging - Socioeconomic determinants of health may complicate behavior change - The availability of services is low, and the effectiveness of low-contact programs is uncertain (e.g., internet-delivered resources)
Footcare	To reduce the risk of further foot complications	Pharmacological: - Antibiotics Nonpharmacological: - Referral to multidisciplinary footcare services - Patient education on footcare - Offloading - Debridement - Revascularization	Low-quality—referral to multidisciplinary footcare services, patient education on footcare	- Footcare ensures regular risk assessment of ulceration and opportunity to modify abnormal risk factors - Referral to multidisciplinary footcare services may reduce the risk of amputation severity, mortality rates and length of hospital stay [86] - A multidisciplinary footcare team with surgical and infection expertise may provide optimal limb salvage treatment [87] - Footcare includes patient education and self-management	- Footcare has no bearing on DN risk - Multidisciplinary footcare services often underperform [88] - There is insufficient evidence to determine if educational strategies reduce the incidence of DFUs and amputations [89] - Patient compliance with self-footcare is often low

**Table 2:** **Advantages and disadvantages of pharmacotherapies for painful diabetic neuropathy.** DN, diabetic neuropathy; IV, intravenous; SNRIs, serotonin and norepinephrine reuptake inhibitors; SSRIs, selective serotonin reuptake inhibitors; TCAs, tricyclic antidepressants; UK, United Kingdom; USA, United States.

Pain Management Strategy	Level of Evidence	Advantages	Disadvantages
Anticonvulsants—pregabalin and gabapentin	Moderate-quality [127,128]	- Anticonvulsants significantly reduce pain in DN [127,128] - Pregabalin is approved for painful DN in the USA and UK [129,130]	- In the USA gabapentin is not approved for painful DN - In the UK there have been recent cases of misuse [131] - Anticonvulsants are associated with tachyphylaxis - Anticonvulsants have a range of side effects (e.g., drowsiness, dizziness, headache, diarrhea and nausea) [130,132] - Pregabalin is linked to infrequent reports of severe respiratory depression [133]
Serotonin and norepinephrine reuptake inhibitors (SNRIs)—duloxetine and venlafaxine	Moderate-quality (duloxetine) [134,135,136,137,138,139,140,141]; Low-quality (venlafaxine) [142]	- Duloxetine significantly reduces pain in DN [134,135,136,137,138,139,140,141] - Duloxetine is approved for painful DN in the USA and UK [129,143]	- SNRIs have similar side effects to anticonvulsants - Sexual dysfunction and sleep problems may be more noticeable [4,143] - Venlafaxine is not approved for painful DN
Tricyclic antidepressants (TCAs) –amitriptyline	Low-quality [144]	- Amitriptyline has benefitted thousands of people with painful DN over the years [144] - Amitriptyline is approved in the UK for neuropathic pain [145]	- Amitriptyline has a range of side effects (e.g., sleep disorders, constipation, sexual dysfunction, arrythmias and postural hypotension) [145] - Amitriptyline is not approved in the USA
Opioids—tramadol and tapentadol	Low-quality [146,147]	- Tramadol significantly reduces pain in DN [148,149] - Tramadol is approved in the USA and UK for moderate to severe pain [129,150] - Tramadol may have a decreased risk for abuse [151] - Tapentadol is approved for neuropathic pain in the USA [129]	- Opioids are linked to problems with misuse and abuse [152] - Opioids have a range of side effects (e.g., dizziness, drowsiness, headache, nausea and constipation) [150] - Tramadol should not be taken in combination with SNRIs/SSRIs [99] - Tapentadol is not approved for neuropathic pain in the UK [153]
Topical analgesics—topical capsaicin	Moderate-quality (8% capsaicin) [154,155]; Low-quality (0.075% capsaicin) [156,157]	- 8% capsaicin significantly reduces pain in DN [154,155] - 8% capsaicin may be more beneficial than anticonvulsants and may have a similar efficacy to duloxetine [154] - 0.075% capsaicin significantly reduces pain in DN [156]	- Some patients may require two to three applications of 8% capsaicin before achieving a treatment response [155] - Topical capsaicin may disturb nociceptive signaling [158] - Topical analgesics only relieve pain in localized areas
Intravenous (IV) medications—IV lidocaine and IV ketamine	Low-quality [159,160,161]	- IV medications significantly reduce pain in DN [159,161]	- IV medications are not currently recommended by clinical guidelines for DN - IV lidocaine may not have long-term effectiveness [159] - IV medications are limited to inpatient use - IV medications have a range of side effects (e.g., sleep disorders, dizziness and nausea) [152,160]

## Data Availability

Not applicable.
